# Statistical framework for validation without ground truth of choroidal thickness changes detection

**DOI:** 10.1371/journal.pone.0218776

**Published:** 2019-06-28

**Authors:** Tiziano Ronchetti, Christoph Jud, Peter M. Maloca, Selim Orgül, Alina T. Giger, Christoph Meier, Hendrik P. N. Scholl, Rachel Ka Man Chun, Quan Liu, Chi-Ho To, Boris Považay, Philippe C. Cattin

**Affiliations:** 1 Department of Biomedical Engineering (DBE), University of Basel, Basel, Switzerland; 2 Institute for Human Centered Engineering (HuCE)-optoLab, Bern University of Applied Sciences, Bern, Switzerland; 3 OCTlab, Department of Ophthalmology, University Hospital Basel, Basel, Switzerland; 4 Department of Ophthalmology, University of Basel, Basel, Switzerland; 5 School of Optometry, The Hong Kong Polytechnic University (PolyU), Hong Kong, PR China; 6 State Key Laboratory of Ophthalmology, Zhongshan Ophthalmic Center, Sun Yat-sen University, Guangzhou, PR China; 7 Moorfields Eye Hospital, London, United Kingdom; 8 Institute of Molecular and Clinical Ophthalmology Basel (IOB), Basel, Switzerland; 9 Wilmer Eye Institute, Johns Hopkins University, Baltimore, Maryland, United States of America; Bascom Palmer Eye Institute, UNITED STATES

## Abstract

Monitoring subtle choroidal thickness changes in the human eye delivers insight into the pathogenesis of various ocular diseases such as myopia and helps planning their treatment. However, a thorough evaluation of detection-performance is challenging as a ground truth for comparison is not available. Alternatively, an artificial ground truth can be generated by averaging the manual expert segmentations. This makes the ground truth very sensitive to ambiguities due to different interpretations by the experts. In order to circumvent this limitation, we present a novel validation approach that operates independently from a ground truth and is uniquely based on the common agreement between algorithm and experts. Utilizing an appropriate index, we compare the joint agreement of several raters with the algorithm and validate it against manual expert segmentation. To illustrate this, we conduct an observational study and evaluate the results obtained using our previously published registration-based method. In addition, we present an adapted state-of-the-art evaluation method, where a paired t-test is carried out after leaving out the results of one expert at the time. Automated and manual detection were performed on a dataset of 90 OCT 3D-volume stack pairs of healthy subjects between 8 and 18 years of age from Asian urban regions with a high prevalence of myopia.

## Introduction

The choroid is a vascular structure located at the posterior part of the uveal tract in the eye, between the relative rigid sclera and the more flexible, light-sensitive retina. The choroid plays a crucial role in the ocular metabolism circulation providing oxygen and metabolites to the outer retina [1, 2]. Its variable thickness depends on factors such as blood pressure, axial length and age [3].

While in adults it has consistently been assessed that the choroidal thickness decreases with advancing age [4–6], studies researching choroidal development during childhood and adolescence led to contradictory conclusions. Subfoveal choroidal thickness was found to be positively correlated with age in Caucasian [7–10], but negatively in Asian children, where the prevalence of myopia is significantly higher [5, 11]. The choroid plays an active role in emmetropization, both by modulation of its thickness to adjust the retina to the optical focus plane (choroidal accommodation) and through the regulation of the scleral growth [12, 13]. Its complex interaction with other tissues as well as its strong dependence on many other factors like blood pressure or diurnal variations, demand a precise and reliable monitoring method [1, 14].

Longitudinal studies of teenagers who develop myopia documented an eye ball elongation. This process is associated with significant thinning of the choroidal thickness in cases of high myopia [15]. Therefore, choroidal thickness, but also choroidal structure, is considered to be an important marker for monitoring myopic progression and for predicting myopia. The main challenge for detecting disease progress is to recognize particular minute changes as early as possible.

Based on optical coherence tomography (OCT) imaging [16] that unveils highly resolved details of the retina and choroid, there are segmentation- and registration-based methods for the detection of temporal changes in the thickness of the choroid (a detailed description of the different state-of-the-art methods follows below). In most cases the evaluation of such methods is performed by an artificially generated ground truth (usually the average of expert segmentations). A major drawback of such an approach is that differences between the manual segmentations cannot be correctly taken into account, when equal weights are given to all expert segmentations during averaging.

In this paper, our primary motivation is to show how to evaluate the performance of a method for choroidal thickness changes detection without generating an artificial average ground truth. We present a validation framework, purely based on common agreement, to assess the detection-performance, using the Williams’ Index [17] as measure. As example of an automatic detection method we use our recently proposed registration-based method CRAR [18]. Additionally, we present an adapted state-of-the-art approach, where an artificial ground truth is created by averaging the results of the remaining experts after leaving one out at the time. In an observational study we examined long-term changes in the choroidal thickness of 90 OCT 3D-volume stack pairs of Chinese subjects between 8 and 18 years of age. For each eye, measurements were collected twice within a period of at least 3 to maximum 14 months.

Our paper’s contributions are: 1) We present a statistical validation framework for automated choroidal thickness changes detection applicable in cases where a real ground truth is not available. 2) We demonstrate the framework’s reliability by evaluating the results of our registration-based algorithm CRAR against those obtained by the experts. 3) We extend the commonly used power analysis approach by leave-one-out cross validation to become an ideal component of our statistical framework. 4) Based on a clinical study with volunteers with a high prevalence of juvenile myopia, we gain insight into possible correlations between time interval (between the measurements) and the choroidal thickness changes measured.

To the best of our knowledge, this is the first time that a statistical validation framework for automated choroidal thickness changes detection combines a method purely based on common agreement with an exhaustive power analysis approach.

### Background and prior work

OCT has become the main contactless, non-invasive method to characterize changes in the corneal, retinal and choroidal structures and to monitor eye growth [16]. Operating in the near infrared range, OCT provides high resolution imaging within a micrometer range and is well established in ophthalmology. Longitudinal studies using OCT imaging offer a unique possibility to gain insight into the dynamics of anatomical changes in the retina and choroid. This leads to an understanding of the mechanisms regulating such processes.

**Fig 1:**
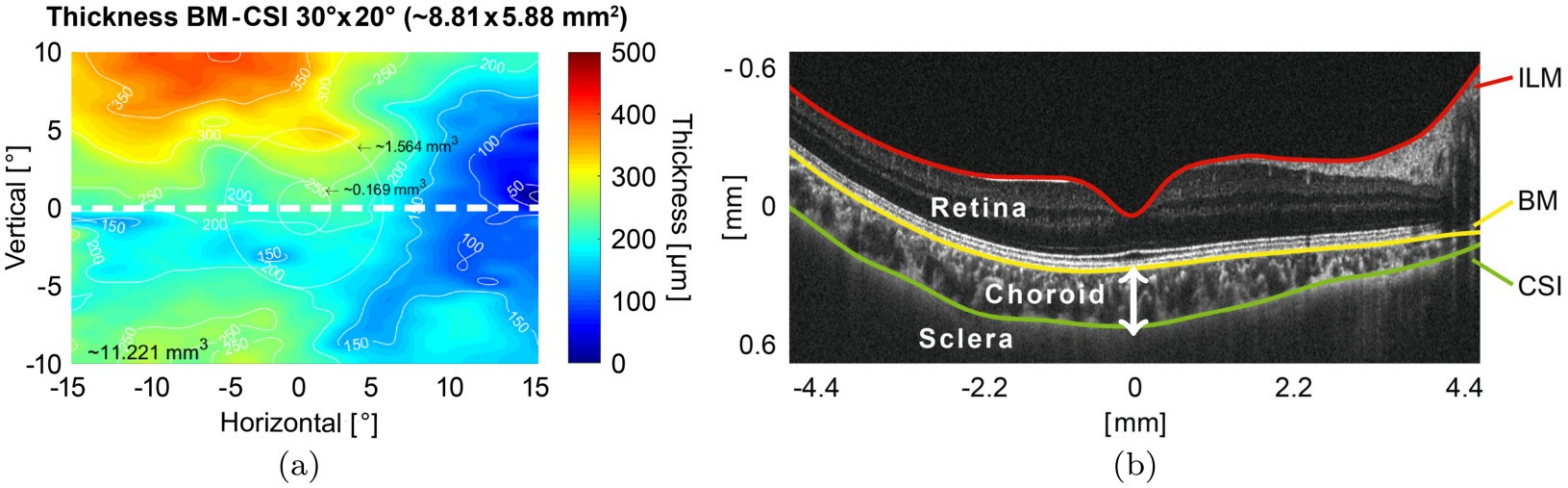
Choroidal thickness map and OCT B-scan with segmented layers. (a) Visualization of the choroidal thickness (BM-CSI) including the choroid’s measured volume of a healthy right eye based on graph search algorithm. Circles indicate the location of the macula. The BM-CSI volume of the whole C-scan is indicated in the bottom left. (b) B-scan, a sagittal cross-section of the posterior eye segment through the retina, choroid and sclera, separated by the layers ILM, BM and CSI (source: Hydra, HuCE-optoLab/BUAS). The image was cut off in the vertical/*z*-direction for better visualization. The full A-scan length is 1.9 mm. The ILM is the Inner Limiting Membrane, while BM and CSI denote the Bruch’s Membrane and Choroid-Sclera Interface, respectively.

The commonly used representation of the thickness of the choroid is the choroidal thickness map, see Fig 1(a), generated from the pre-segmented data of a 3D-volume stack (C-scans) consisting of adjacent sagittal tomograms (B-scans). For every depth scan (A-scan) the difference between two segmentation-planes at this lateral location is calculated and represented. The choroidal thickness is defined as the vertical distance (along the A-scans/in *z*-direction) between the Bruch’s Membrane (BM) and the Choroid-Sclera Interface (CSI), see Fig 1(b). Segmentation is frequently performed as a manual task to determine the ground truth from OCT-measurements. Due to the lack of alternative high precision in-vivo measurement methodologies, signals are typically compared to their histological equivalent. This proves to be notoriously difficult, subjective, and unreliable in view of the large amount of data points and the weak signals that are frequently hard to interpret for the human observer [14]. Even the intra-observer reproducibility is relatively low and with novel algorithms that excel in this regard a different approach to verify the reliability has to be taken. Automated detection of noisy and speckled OCT-images at the low-signal-end of the depth scan already has a long history and usually focuses on segmentation by delineating borders that are associated with large scale changes of the refractive index, or by determining tissue texture appearance.

Among the current approaches for detecting choroidal boundaries, graph search based segmentation methods represent a state-of-the-art [19–21]. However, their performance is limited by the low contrast of the choroidal boundaries, the inhomogeneity of the choroid’s texture and great variation of the choroidal thickness [2]. Recently, a segmentation algorithm was presented, which combines a robust contour-detection method with a graph search, based on a novel weighting scheme [22]. However, the reliability of this method depends strongly on the choice of nodes and weights. In [23], CSI segmentation was performed using an improved graph search algorithm with curve smooth constraints. However, this approach was especially developed for Cirrus HD-OCT (High Definition) and still needs to be extended to other OCT devices. The use of a convolutional network architecture, where an optimal graph-edge weight can be learned directly from raw pixels, was proposed in [24]. However, this approach requires a huge amount of training data (approximately 1000 manually segmented B-scans) from the experts. In [25] the authors presented a method for unsupervised learning to identify anomalies in imaging data as candidates for pathological markers. However, it still has to be proven that the method is really able to recognize very small changes, for example in an early stage of a disease. Despite progress in image processing the use of single frame segmentation is inherently difficult, especially in longitudinal clinical studies where successive imaging sessions can strongly vary in signal quality.

In order to circumvent this limitation, we recently proposed CRAR, a method to detect early **C**horoidal changes using piecewise rigid image **R**egistration and eye-shape **A**dherent **R**egularization [18]. Our method is a registration-based approach specifically developed for longitudinal studies, allowing to overcome critical problems like low contrast, loss of signal and the presence of artifacts, which are yet unsolved by most segmentation-based methods for the detection of minute choroidal changes. It needs to be emphasized that the aim of CRAR is not to localize the exact position of the CSI, i.e. the exact cutting line between choroid and sclera, but to figure out the displacement in anterior-posterior/*z*-direction of its surrounding area within a specific time interval. Based on the resulting distortion fields, the use of a roughly localized CSI borderline is already sufficient to extract the corresponding volume changes.

In a previous paper [18] we already demonstrated CRAR’s robustness regarding noise by testing the method on scan-rescans. As by scan-rescan no changes occur except the noise, the detected displacement field must be close to zero. By inducing synthetic deformations in the area of the CSI, we attested CRAR’s applicability on follow-ups as well as its ability to detect changes as small as 5 μm in the thickness of the choroid [18]. For more details about CRAR the reader is also referred to the S1 Appendix.

## Method

**Fig 2:**
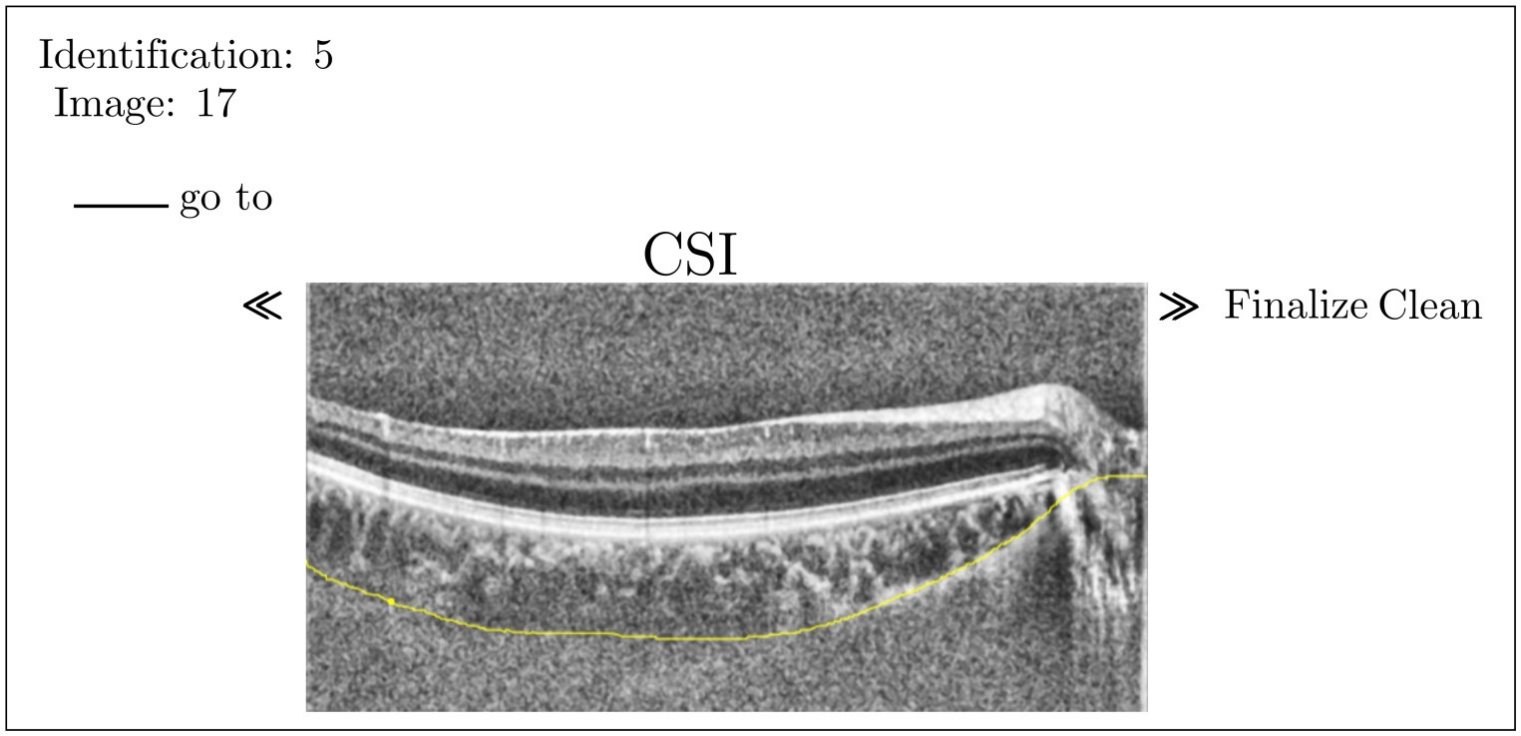
Sample screen of our online tool for manual expert segmentation. According to the consensus between the experts, interconnecting tissues and vessels inside the sclera were ignored while the yellow segmentation line was continued on the side of the optical nerve horizontal. The pre-processing (filtering and histogram equalization) for better contrast during the task was activated in this case by the expert.

To deal with the lack of valid ground truth information, we compare the results achieved by an automatic technique with those of a group of human experts, see Fig 2. In this context, one assumes that human raters hold some prior knowledge of a “mental” ground truth that is reflected in their manual tracings [26, 27]. Human rater accuracy and variability is taken into account by measuring the similarity between the expert and automatic segmentation [28]. Since a solid ground truth does not exist, the most natural solution is an evaluation based on common agreement.

The key idea is the following: we define a method *X* to be at level with a group {*Y*_*i*_} of experts if *X* agrees with each expert *Y*_*i*_ at least as often as the experts {*Y*_*i*_} agree among themselves. In other words, with the help of an agreement index, we show that the agreement rate between algorithm *X* and each expert is at least as high as within the expert group itself. First, we apply the similarity measures commonly used for evaluations of segmentation results. Since we are not looking for a surface but a contour (the CSI, the lower boundary of the choroid), we need a more appropriate measure for contours. To show how to proceed when working with a registration approach, where the outcome is the displacement of the contour line during the time interval between the two measurements (and not the contour itself), we use our previously published algorithm CRAR as example for method *X*. In this case, the displacement of the contour line is a very natural and intuitive metric to be used. The algorithm’s performance is evaluated with an agreement test by comparing the displacements detected by CRAR with those of the experts. As an agreement measure we opt for the Williams’ Index [29] and demonstrate that the algorithm’s performance is independent of the chosen metric. In addition, for comparison with a state-of-the-art evaluation procedure, we conduct a paired t-test using an artificially generated ground truth.

### Intra-rater coefficient

In order to quantify the reliability of each expert in segmenting the same image we introduce the corresponding Intra-Rater Coefficient. Let us consider a set of *S* images, namely OCT B-scans of size *m* × *n* pixels. Each image has to be segmented thrice by an expert. Considering the *s*^th^ image segmented by expert *j*, the deviation $\Delta E_{j}^{s}=E_{j}^{s}-{\bar{E}}_{j}^{s}$ of any one of the three segmentations $E_{j}^{s}$ from their average ${\bar{E}}_{j}^{s}$ is calculated. The number #counts(*j*, *l*) of all such deviations, which are within a given tolerance interval [−*l*, *l*], i.e.

$$\#\text{counts}\left(j,l\right)=\#\{\Delta E_{j}^{s}|-l\leq \Delta E_{j}^{s}\leq l\},\;\text{where}\;\;s\in \{1,\ldots ,S\},$$

leads to the definition of the Intra-Rater Coefficient IRC_*j*_ of expert *j*

$${\text{IRC}}_{j}=\frac{\#\text{counts}(j,l)}{3nS}.$$ (1)

Here, 3*nS* denotes the total number of pixels to be segmented by each expert and *l* is the margin of tolerance set for the manual segmentation (in our case *l* = 20 μm or 5 pixels).

Now, we try to get a feeling for the value of this coefficient. It can be shown that a rater that repeats the segmentation of the CSI at random will achieve an IRC of at most 0.2. Such a rater can be simulated by inducing, in the manually annotated scans, synthetic B-spline deformations with randomly chosen coefficients for the linear combinations of their basis functions. In order to attest reliability to the manual segmentations, we aim to achieve IRC values of at least 0.70. This value corresponds to a variance in the segmentations of one rater of approx. 25 μm.

### Williams’ index

According to Williams [17] and [29] we propose an agreement index giving an answer to the following question: given a group of *r* ≥ 3 raters labeling a finite set of pixels, does rater *j* agree with a group of experts in the same way as the group members agree among each other? If this is the case, the index of agreement is set equal to 1.

Let $E_{j},E_{j^{\prime }}$ and $E_{j^{\prime \prime }}$ be the results of all the manual segmentations by the experts *j*, *j*′ and *j*″, the Williams’ agreement Index (WI) of expert *j* is defined as:

$$WI_{j}=\frac{\left(r-2\right)\sum_{j^{\prime }\neq j}^{r}s\left(E_{j},E_{j^{\prime }}\right)}{2\sum_{j^{\prime }\neq j}^{r}\sum_{j^{\prime \prime }\neq j}^{j^{\prime }-1}s\left(E_{j^{\prime }},E_{j^{\prime \prime }}\right)},$$ (2)

where $s\left(E_{j},E_{j^{\prime }}\right)\in \left[0,1\right]$ provides a quantification for the similarity between the predictions of rater *j* and *j*′ for all pixels (for more details about $s(E_{j},E_{j^{\prime }})$ see the next subsection). The ratio derived is compared to the value of 1. If this index is greater than 1, it can be concluded that rater *j* agrees with the other raters at least as often as they agree with each other [29]. Otherwise, the rate of agreement obtained between rater *j* and the group of raters is smaller than the rate of agreement within the group of raters.

### Similarity measures

The main underlying principle of our evaluation is the definition of agreement between a method *X* (here CRAR) for automated detection of choroidal thickness changes and a group of experts doing manual segmentations. The agreement of two experts is defined as the similarity between their respective segmentations. An intuitive similarity measure for the comparison between our algorithm and manual segmentations is the difference between the automatically detected changes and the difference between manual segmentations by experts at different times. The evaluation framework is established by first calculating the WI using the common similarity measures for segmentation, i.e. the Dice Coefficient (DC) and the Jaccard Similarity Coefficient (JC). Such similarity measures are typically applied to survey the segmentation of surfaces.

**Fig 3:**
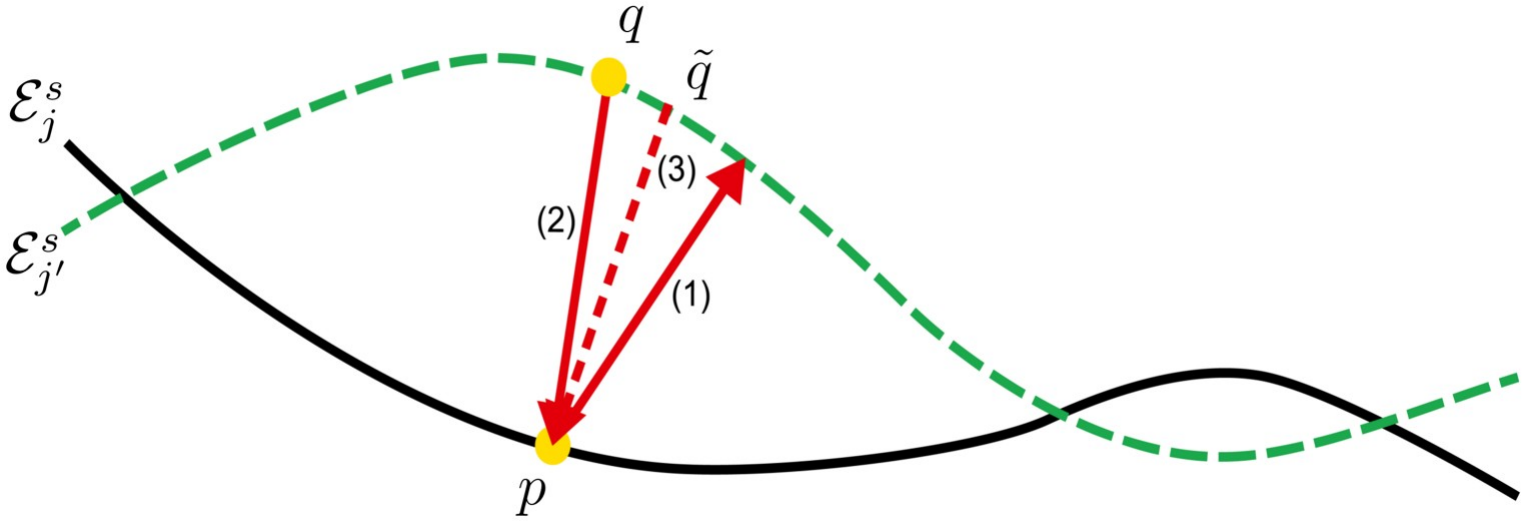
The calculation of the similarity measure BLD. First, the minimum “forward” distance $d_{\text{min}}\left(p,E_{j^{\prime }}^{s}\right)$ between the point $p\in E_{j}^{s}$ and the contour $E_{j^{\prime }}^{s}$ is determined, here marked as (1). Second, among all the points *q* on $E_{j^{\prime }}^{s}$ with a “inverse” minimum distance $d_{\text{min}}^{-1}\left(q,E_{j}^{s}\right)$, those are selected whose minimal distance is found at the point *p*. Here, *q* and $\tilde{q}$ are the candidates, with the corresponding distances denoted by (2) and (3). Then, the maximum distance among the candidates, in this case (2), is chosen as $d_{\text{max}}^{-1}\left(E_{j^{\prime }}^{s},p\right)$. Finally, $\text{BLD}(p,E_{j^{\prime }}^{s})$ is defined as the maximum between $d_{\text{min}}\left(p,E_{j^{\prime }}^{s}\right)$ and $d_{\text{max}}^{-1}\left(E_{j^{\prime }}^{s},p\right)$, in this example (2). For more details see [30].

**Fig 4:**
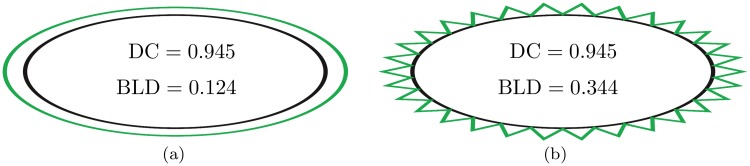
The robustness of the BLD in comparison to the DC for contour recognition. Here, the value of the surface delimited by the green contour is the same in both cases: (a) The region which should be recognized is an ellipse (black). (b) While the original contour was not recognized well at all, the DC for such a segmentation has yet the same high value as in (a). Using the BLD, we achieve a fairer evaluation of the segmentation, as the bad contour detection is taken into account and penalized with a higher value of the BLD (which corresponds to a minor similarity).

As our task is to recognize the CSI, the lower contour of the choroid, we need a more appropriate similarity measure. For this reason we opt for the Bidirectional Local Distance (BLD, see Fig 3, for more detail see [30]), a more robust and conclusive similarity measure for surfaces than the DC and JC, as shown in Fig 4.

Let *S* be the number of B-scans to be segmented by each expert and let $E_{j}={\left\{E_{j}^{s}\right\}}_{s=1}^{S}$ and $E_{j^{\prime }}={\left\{E_{j^{\prime }}^{s}\right\}}_{s=1}^{S}$ denote the segmentations done by the experts *j* and *j*′ respectively. We define

$$\text{BLD}\left(E_{j},E_{j^{\prime }}\right)=\sum_{s=1}^{S}\sum_{p\in E_{j}^{s}}\frac{\text{BLD}(p,E_{j^{\prime }}^{s})}{n\underset{p\in E_{j}^{s}}{\text{max}}\left\{\text{BLD}\left(p,E_{j^{\prime }}^{s}\right)\right\}},$$ (3)

where

$$\text{BLD}\left(p,E_{j^{\prime }}^{s}\right)=\text{max}\left\{d_{\text{min}}\left(p,E_{j^{\prime }}^{s}\right),d_{\text{max}}^{-1}\left(E_{j^{\prime }}^{s},p\right)\right\},$$

and $d_{\text{min}}\left(p,E_{j^{\prime }}^{s}\right)$ corresponds to the minimum distance from a point *p* on the reference $E_{j}$ to the test contour $E_{j^{\prime }}^{s}$, while

$$d_{\text{max}}^{-1}\left(E_{j^{\prime }}^{s},p\right)=\underset{q\in E_{j^{\prime }}^{s}}{\text{max}}\{d_{\text{min}}\left(q,E_{j}^{s}\right)|d_{\text{min}}^{-1}\left(q,E_{j}^{s}\right)=||q-{p||}_{2}\}$$

denotes the maximum inverse distance at *p* on $E_{j}^{s}$, as illustrated in Fig 3.

Although BLD is more robust than Dice and Jaccard for the detection of contours, (see Fig 4) it is, like Dice and Jaccard, not suited for the algorithm we present. As mentioned above, this algorithm is registration- but not segmentation-based, and its result is the displacement field over time of the entire border area between sclera and choroid (including the exact CSI, which is very difficult to localize). Therefore, the algorithm does not provide contours and thus, BLD, Jaccard, and Dice are not suitable as metric because we compare neither overlapping surfaces nor contours.

Consequently, we need to introduce an appropriate metric for our task, as of now diffZ (see Eq (4)) consisting of the difference between the detected displacements of the algorithm and the differences between the expert segmentations at different times, i.e.

$$\text{diffZ}\left(\Delta E_{j},\Delta E_{j^{\prime }}\right)=\frac{\sum_{s=1}^{S}\sum_{i=1}^{n}|\Delta E_{j}^{s}\left(i\right)-\Delta E_{j^{\prime }}^{s}\left(i\right)|}{mnS},$$ (4)

where $\Delta E_{j}={\left\{\Delta E_{j}^{s}\right\}}_{s=1}^{S}$ and $\Delta E_{j^{\prime }}={\left\{\Delta E_{j^{\prime }}^{s}\right\}}_{s=1}^{S}$ represent the displacements in anterior-posterior/*z*-direction detected by the expert *j* and *j*′ respectively, and are computed as the difference between the segmentations of the second measurement and those of the first one. The metric diffZ provides a value between 0 and 1, denoting the normalized difference (or, in other words, the amount of disagreement) between the detected displacements of algorithm and one expert, or, between any two experts, respectively. As a result, 0 means “no difference” (or, maximal agreement at each point) and 1 “maximum difference” (or, complete disagreement), respectively. The metric diffZ is especially suitable for the evaluation of registration-based algorithms such as CRAR, in which no contours are shown. At the same time, it can also be applied in longitudinal studies to evaluate segmentation-based algorithms, which aim at the segmentation of the CSI. In this case, diffZ can be defined for the algorithm as the difference between the segmentations of two measurements performed within a time interval.

### Power analysis (paired t-test)

As an additional component to the presented validation framework, we now perform an extended power analysis. Let $\Delta E={\left\{\Delta E_{j}^{s}\right\}}_{j=1,s=1}^{r,S}$ denote the total of all displacements detected by all experts. For each *j* ∈ {1, …, *r*} the artificial ground truth

$${\bar{G}}_{j}=\frac{1}{r-1}\sum_{j^{\prime }\neq j}^{r}\Delta E_{j^{\prime }},\;\;\;\text{for}\;\;\text{all}\;\;\Delta E_{j^{\prime }}\in \Delta E\backslash \left\{\Delta E_{j}\right\},$$ (5)

is defined by leaving out the results of expert *j* and calculating the average of the displacements detected by the remaining experts.

Let *X* denote the displacements detected by the algorithm. For each expert *j* a paired t-test is done to compare the errors $X_{j}=X-{\bar{G}}_{j}$ and $Y_{j}=\Delta E_{j}-{\bar{G}}_{j}$. In other words, after defining an artificial ground truth, we compare the difference in the errors of both algorithm and experts in their detection of choroidal thickness changes. Thus, we test the null hypothesis that the pairwise differences *X*_*j*_ − *Y*_*j*_ come from a normal distribution with mean equal to 0 at the *α* = 0.01 significant level. In order to reject the null hypothesis, the result of the *p*-value must be smaller than *α*. While a *p*-value shows whether an effect exists, it will not reveal the size of the effect (it might be, that a smaller *p*-value has occurred only on the basis of a large sample size [31]). This is why, we report both statistical (the *p*-value) and substantive significance (effect size). Using the Cohen’s distance *d* between both datasets, the effect size can be determined by calculating the mean difference between the two groups *X*_*j*_ and *Y*_*j*_, and then dividing the result by the pooled standard deviation $\sqrt{S}$, i.e.

$$\text{Cohen}\prime s\;d_{j}=\frac{\mu \left(X_{j}\right)-\mu \left(Y_{j}\right)}{\sqrt{S}},\;\;\;\text{where}\;S=\frac{\left(\right|X_{j}|-1)\cdot \sigma^{2}\left(X_{j}\right)+\left(\right|Y_{j}\left|-1\right)\cdot \sigma^{2}\left(Y_{j}\right)}{|X_{j}\left|+\right|Y_{j}|-2},$$ (6)

where |*X*_*j*_| and |*Y*_*j*_| denote the sample sizes of *X*_*j*_ and *Y*_*j*_ respectively, while *σ*(*X*_*j*_) and *σ*(*Y*_*j*_) are their standard deviations. The necessity of this “leave-one-out” power analysis lies in the fact that, in our case, an artificially generated ground truth can only be represented in the form of a matrix, as to every OCT B-scan pair a corresponding ground truth for comparison has to be generated. This results for ${\bar{G}}_{j}$ in a matrix of size *n* × *S* corresponding to the ground truth for the entire dataset. As a result, the values of the standard deviations for $X-{\bar{G}}_{j}$ vs. $\Delta E_{j}-{\bar{G}}_{j}$ are different from those for *X* vs. $\Delta E_{j}$. Consequently, the values of the effect size, quantified by Cohen’s *d*, change as well.

## Material

### Subjects

Ninety OCT 3D-volume stack pairs of Chinese subjects, aged 8-18 and stemming from urban regions with a high prevalence of myopia, have been analyzed. Healthy subjects with good distant and near vision (monocular corrected visual acuity was equal or better than LogMAR 0.00), but no systemic and ocular diseases, ocular trauma or surgery, were recruited. For the subjects aged 8-13, the spherical refractive errors were -1.00D to -5.00D and cylindrical power was not more than -1.50D. For the others, the spherical refractive errors were +0.75D to -3.00D and the cylindrical power was not more than -1.00D. Written consent was obtained from both volunteers and, when necessary, their parents. The study protocol was approved by the Human Subjects Ethics Sub-committee of The Hong Kong Polytechnic University and was conducted in adherence to the tenets of the Declaration of Helsinki.

### Data acquisition

The volunteers were measured twice at different times: half of them their left eye, the other half their right one. This resulted in 180 OCT volume stacks, each consisting of 25 B-scans of 500 × 768 pixels. The pixel spacing in nasal-temporal/*x*- and superior-inferior/*y*-direction were set to 11.46 μm/pixel and to 245 μm/pixel respectively, the one in anterior-posterior/*z*-direction was set to 3.87 μm/pixel. The volunteers were divided into three groups: the first group was measured a second time after 3 months, the second after 8 months, the third after 14 months.

The images were acquired by an eye-tracking dual-wavelength OCT system operating simultaneously at the 870 and 1075 nm bands. This system was developed at the HuCE-optoLab at the Bern University of Applied Sciences in Biel before it was transferred and setup at the Hong Kong Polytechnic University’s School of Optometry. For technical details on the OCT system used we refer to the S2 Appendix.

### Manual expert segmentation

Six ophthalmologists were recruited as experts. The experts received access to a Java-based online tool with which they could draw the CSI line, using either the mouse (PC) or a pen (tablet), see Fig 2. The experts could chose to segment the CSI without processing or to activate a pre-processing consisting of a histogram equalization and an average filtering. In this evaluation step the focus lay on both the intra-rater reliability of each individual expert and the agreement among the experts.

The 90 volume stack pairs were randomly distributed among the six experts, in such a way that each expert got exactly 50 volume stack pairs and each pair was assigned to at least three different experts for CSI segmentation. This allowed to test the agreement between the experts. From each volume stack pair, eight B-scan pairs were chosen for manual expert segmentation of the CSI, three in the lower (no. 1, 3 and 6), three in the middle (no. 11, 13 and 16) and two in the upper region (no. 21 and 23). To test the intra-rater reliability, each expert unknowingly received three times the same scan pair. The number of lines to be segmented by each expert was 2400 (8 scan positions per volume stack × 2 measurements per volunteer × 50 volume stacks × 3 repetitions). Calculating a time of approx. 9 sec per line, this adds up to 6 h per expert.

### Manual segmentation (consensus)

Manual segmentation was done according to the following mutual consensus: the lower border of the choroid was drawn without taking into account the interconnecting tissues that appear as humps on top of the slowly varying baseline. Also the shadow artifacts that are cast by the retinal and choroidal vasculature were ignored. Vessels inside the sclera were also disregarded. It was agreed that once the CSI came to an end near the optical nerve, the segmentation line would be continued in the same direction and with the same slope, as depicted in Fig 2.

## Results and discussion

**Fig 5:**
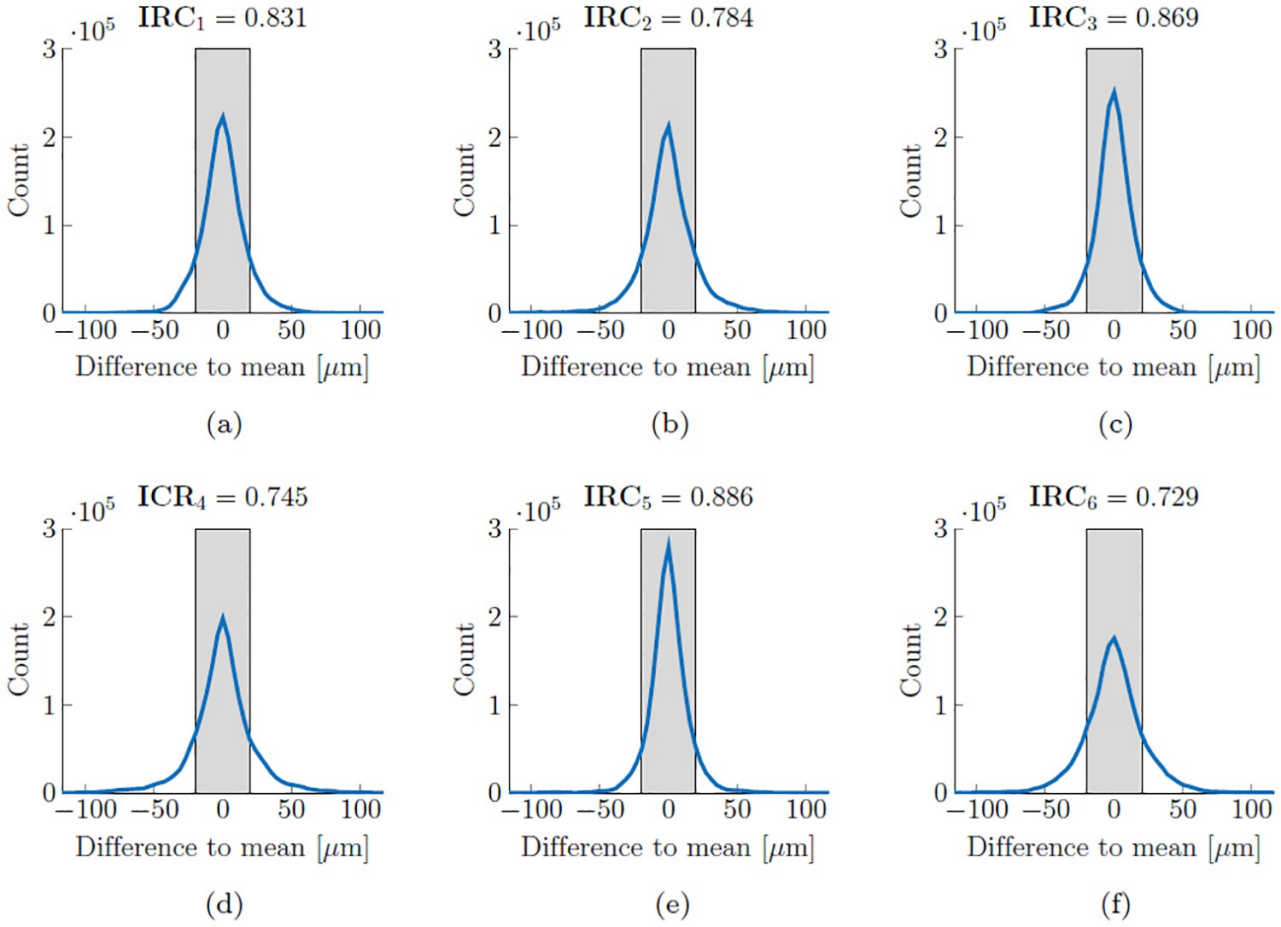
The representations of the intra-rater reliability of experts 1–6 ordered from (a)–(f). At every pixel position, the difference to the average value of the three available segmentations per rater is calculated. If its absolute value is smaller than a predefined threshold (here set to 20 μm represented by the grey area) then it is counted, i.e. the prediction is considered reliable. Therefore, the narrower and higher the curve, the more reliable the segmentation by the corresponding expert is. The number of counts found within this range is divided by the total number of segmentation points graded by the corresponding expert. By the obtained normalized value IRC_*j*_ we define the Intra-Rater Coefficient to quantify the reliability of the *j*^th^ expert.

**Fig 6:**
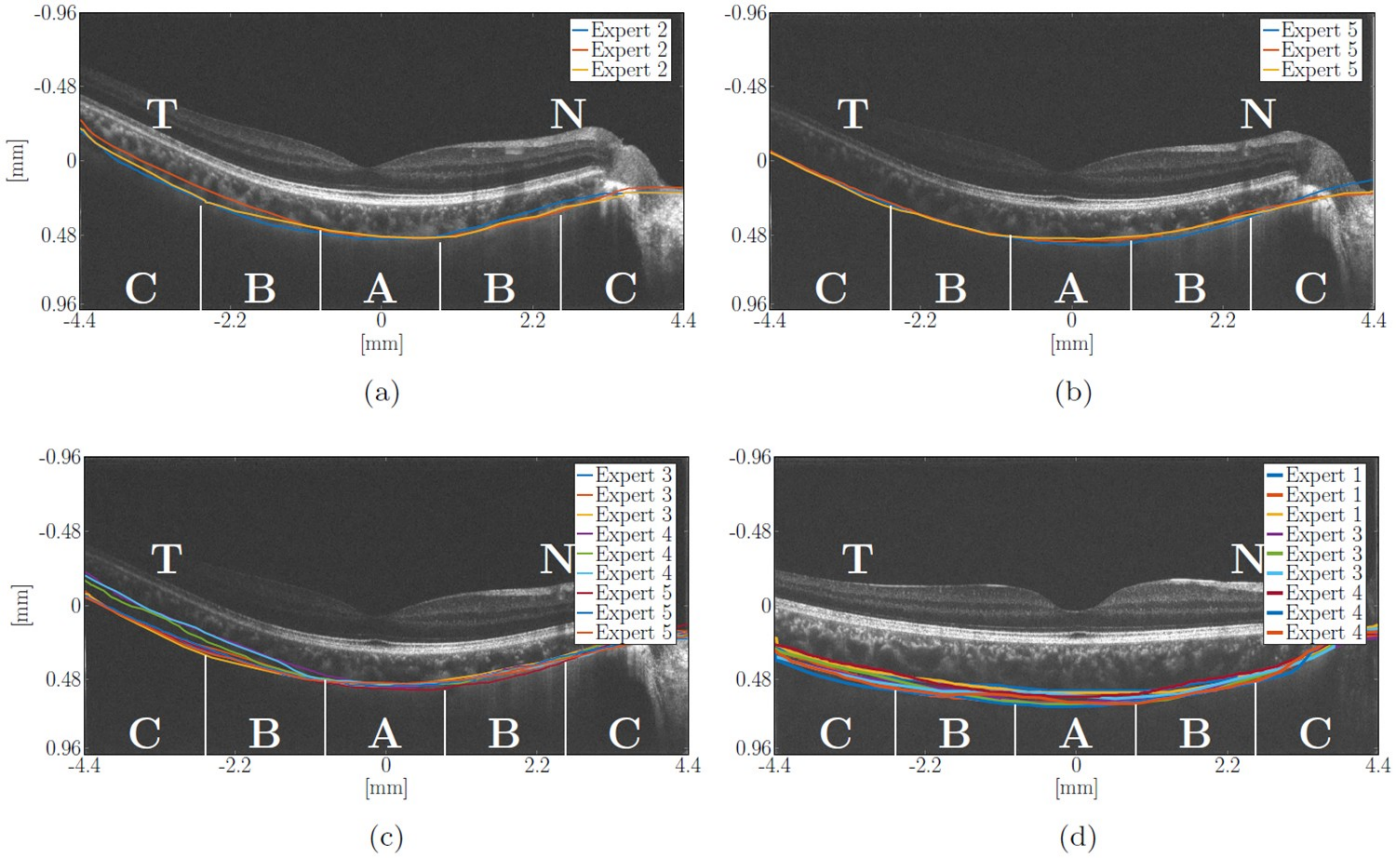
Examples of manual expert segmentation (consistent and less consistent with each other). Top: Repeatability of (a) expert 2 and (b) expert 5 when segmenting the CSI. Bottom: Comparison of segmentations by (c) experts 3, 4 and 5 and (d) experts 1, 3 and 4. The choroidal area is subdivided in nasal (N)-temporal (T)/*x*-direction into five equidistant regions (patches) symmetrically around the foveal center: A (foveal region), B (parafoveal region), and C (perifoveal region). Here only cases of right eyes are depicted.

Fig 5 shows the intra-rater reliability (repeatability) of the results of each expert by having the same image segmented thrice. The six experts together show an averaged standard deviation of ±24.73 μm in rating the same image. It is remarkable, that the experts are often inconclusive about the position of the CSI in the temporal para- and perifoveal region opposite to the ocular nerve, see later Fig 6(c) and 6(d). This is due to the low contrast in the image acquisition in this area of the choroid which makes a clear identification of the CSI difficult. The mean IRC value achieved by the experts was 0.807, which is to be considered as a good result. It has to be pointed out that the lowest scores were reached by those experts who probably had experienced difficulties in handling the tool. The first version of our online tool has been continuously optimized based on the feedback of the raters. Despite the challenges, the IRC scores were almost as good as we wished for.

**Fig 7:**
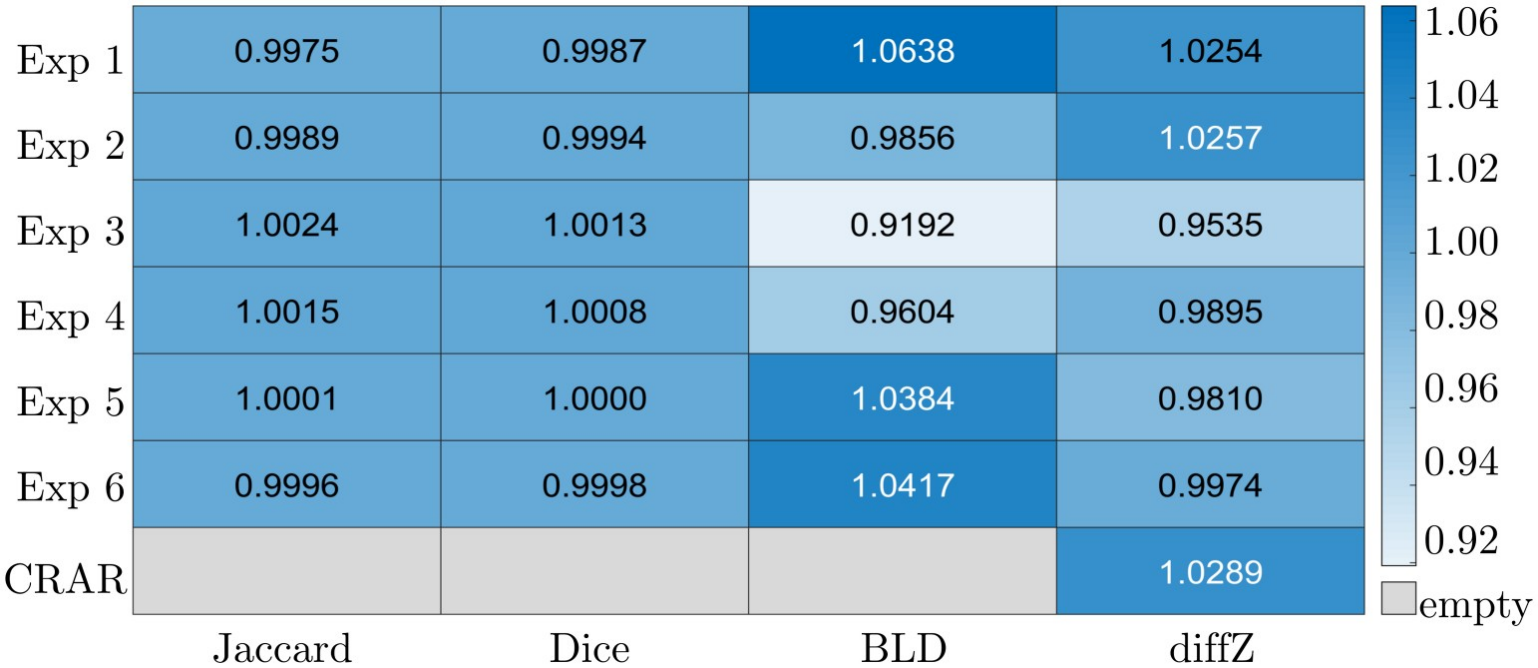
The values of the WI calculated for the algorithm and the experts group. As similarity measures Jaccard, Dice, BLD and diffZ are used.

Fig 7 presents the values of the WI calculated between each individual expert and the other members of the group for all the images (using DC, JC, BLD and diffZ as metric). When we compare the segmentations performed by the experts, the results show an average WI of 0.9992 ± 0.0221. The weak scattering of the WI demonstrates that its value does not depend on the choice of the similarity measure. Fig 7 shows that there are no relevant differences between the values of the WI whether they are calculated on the base of diffZ or the other three metrics, so Dice, Jaccard and BLD are not indispensable to this kind of evaluation, and therefore no longer needed. This justifies the use of diffZ for our case even more. The comparison between CRAR and the experts group shows that CRAR’s predictions match those of the experts (the WI is greater than 1). To exclude that one expert influences the value of the WI much more than another, the WI is recalculated by omitting one expert at a time. The calculation of the WI between CRAR and the remaining five experts after leaving-one-out gives values in the range of [1.0126, 1.0393].

**Fig 8:**
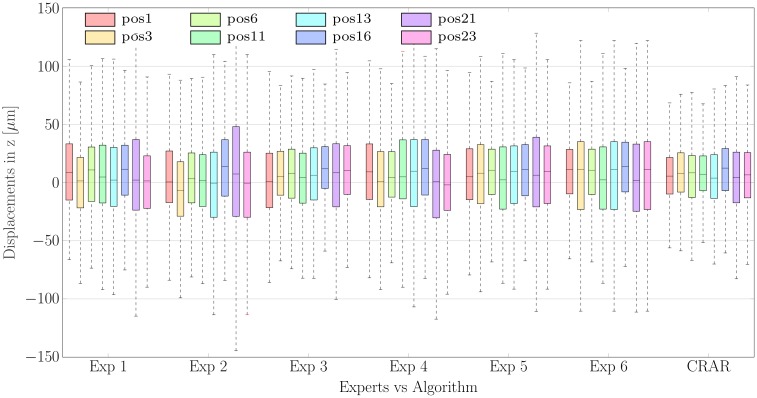
The average displacements of the CSI grouped by experts and algorithm. The results are obtained by manual segmentation by the six experts and by CRAR (subdivided into the B-scan positions 1, 3, 6, 11, 13, 16, 21 and 23).

Fig 8 shows the variability at each scan position grouped by expert. As expected, the displacements detected by CRAR (4.29 ± 23.73 μm, see the far right hand side of Fig 8) are within the range of those detected by the experts (5.47 ± 39.45 μm) but with a smaller variance. This is due to the fact that our automated algorithm guarantees perfect repeatability (IRC = 1), unlike the human rater.

**Fig 9:**
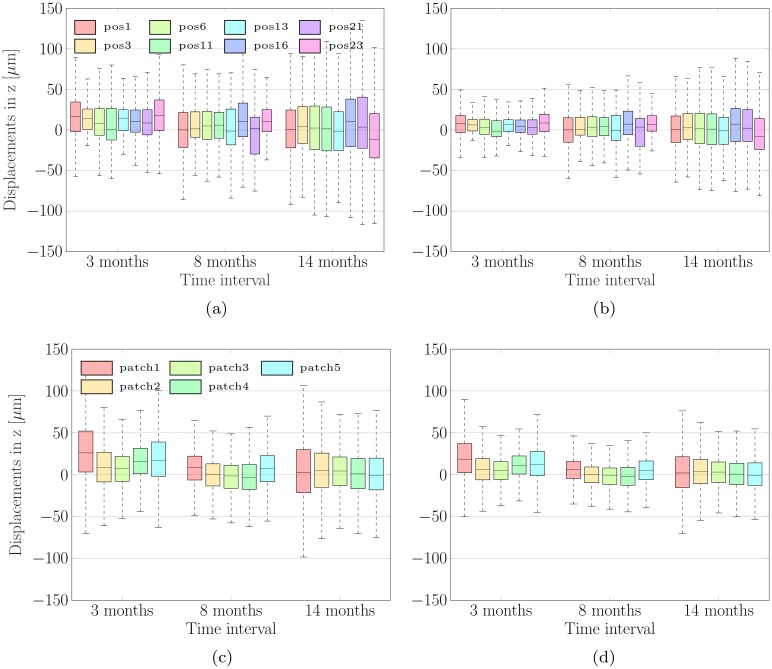
The average displacements of the CSI grouped by time intervals. Above: The average displacements of the CSI detected by the expert group (a) and by CRAR (b) grouped by time intervals between the two measurements and subdivided into eight scan positions. Below: The average displacements of the CSI detected by the expert group (c) and by CRAR (d) grouped by time interval and subdivided in nasal-temporal/*x*-direction into five equidistant regions C-B-A-B-C (patches) symmetrically distributed around the foveal center, see Fig 6.

Fig 9(a) and 9(b) show the total variability of all displacements detected by the experts and the algorithm, respectively, grouped by time interval between the two measurements. The average displacements measured using CRAR are 1.41 ± 16.23 μm in the case of images captured within a time interval of 3 months. For the other two time intervals of 8 and 14 months, the average changes are 4.88 ± 22.63 μm and 6.12 ± 29.32 μm, respectively. These results are still consistent with those of the experts, i.e. 1.76 ± 26.72 μm, 5.67 ± 32.48 μm and 7.62 ± 39.15 μm but with smaller variations. These results are also summarized in Table 1 for better data visualization. During each time interval between measurements the choroidal thickness increases, as is to be expected for growing tissue. This finding supports the hypothesis which several studies [7–10] formulate: the increase in thickness of the choroid is a normal feature of eye growth from early childhood to adolescence. Such a thickening of the choroid could relate to changes in the structure and associated physiological demands of the outer retina occurring during the eye’s natural development, and/or to its role as sclera growth regulator [13].

**Table 1:** The averaged vertical displacements Δ*z* measured at different time intervals.

mean Δ*z* in [μm] grouped by time interval			
	3 months	8 months	14 months
Experts	1.76 ± 26.72	5.67 ± 32.48	7.62 ± 39.15
CRAR	1.41 ± 16.23	4.88 ± 22.63	6.12 ± 29.32

The table notes the detected temporal changes depicted in Fig 9(a) and 9(b) averaged per time interval between the two measurements.

On the other hand, the thickening of the choroid seems to slow down as time goes by, while the scattering of the data increases. The slower progress in thickening could be related to the high prevalence of Chinese children to become myopic (≈ 80% in the age 13–15). It could also be a sign that a disproportionate elongation of the eye-ball is taking place and must be compensated by slowing down the growth or, in case of myopia, even an actual thinning of the choroidal thickness at a relatively earlier age. The slowing down process of the thickening could also be the result of the natural “plateau” effect of the growth [10] in thickness of the choroid, in analogy to that of the body size. In other words, it appears that the thickness of the choroid increases in early childhood, reaching a peak between 10 and 20, and then exhibits a gradual decrease into older adulthood [8, 10]. However, it has to be noted that these assumptions are based on cross-sectional studies and, thus, the same subjects have not been observed regularly during longer periods of time. Therefore, before it can be generalized that the thickness of the choroid increases from early childhood to adolescence, further longitudinal research is ongoing, in which the subjects are being measured regularly and more frequently. The time interval related increase in scattering of the detected displacements is a natural consequence of the diversity in choroidal changes, which, most likely, vary from subject to subject.

As mentioned above the results of CRAR are in line with those of other clinical studies: according to [9, 10] changes in the choroidal thickness appear to rapidly increase in early childhood (age 4–7, mean increase of 30 ± 15 μm within a time interval of 18 months), followed by a plateau in thickness change in the older age groups examined (age 10–13, mean change 13 ± 22 μm in 18 months). These aspects are reflected in our results and can be explained by the fact that the subjects that took part in this study are in an age phase, in which the choroid is still growing, but not as intensively as in early childhood (4–6 years old).

Fig 9(c) and 9(d) show the detected temporal changes grouped by time intervals and subdivided in nasal-temporal/*x*-direction into five equidistant regions C-B-A-B-C (patches) symmetrically distributed around the foveal center: A (foveal region), B (parafoveal region), and C (perifoveal region), see Fig 6. Here the mean changes detected by CRAR in the regions C-B-A-B-C (from the temporal (T) to the nasal (N) location in the case of a right eye, as shown in Fig 6, and in the opposite direction in the case of a left eye): 6.75 ± 27.12 μm (C), 4.07 ± 19.23 μm (B), 1.21 ± 13.57 μm (A), 4.23 ± 17.01 μm (B) and 5.18 ± 20.38 μm (C), respectively. These values are in the range of those detected by the experts: 9.95 ± 37.18 μm (C), 5.13 ± 28.53 μm (B), 1.81 ± 16.21 μm (A), 4.17 ± 22.37 μm (B) and 6.27 ± 26.01 μm (C), respectively. These results are also summarized in Table 2 for better data visualization.

**Table 2:** The vertical displacements Δ*z* averaged per choroidal subregion.

Δ*z* in [μm] averaged per choroidal subregion C-B-A-B-C					
	C(perifoveal)	B(parafoveal)	A (foveal)	B (parafoveal)	C(perifoveal)
Experts	9.95 ± 37.18	5.13 ± 28.53	1.81 ± 16.21	4.17 ± 22.37	6.27 ± 26.01
CRAR	6.75 ± 27.12	4.07 ± 19.23	1.21 ± 13.57	4.23 ± 17.01	5.18 ± 20.38

The table notes the detected temporal changes depicted in Fig 9(c) and 9(d) averaged per choroidal subregion C-B-A-B-C (i.e. the five equidistant patches symmetrically distributed around the foveal center).

According to [32, 33], the results show a prominent choroidal thickening in more peripheral regions and, in general, it can also be concluded that the first changes occur in the periphery rather than in the center. The error of CRAR is significantly lower than for the remaining five experts after leaving out one expert segmentation $E_{j}$ at a time (*p*-value<0.01) with medium effect size (Cohen’s *d* in the range of 0.41 and 0.49). This emphasizes the superior performance of CRAR in detecting choroidal thickness changes in comparison to those of the experts group.

The power analysis for different time intervals showed a small but not irrelevant effect size, and thus a change in the thickness of the choroid, which cannot be neglected. This supports the tendency mentioned above. For example, comparing the results for the time intervals of 3 and 14 months, we obtained a Cohen’s *d* of 0.20 for the experts and 0.25 for the algorithm.

## Conclusion

In this paper, we presented a statistical framework for validation of choroidal thickness changes detection without ground truth. Using the William’s Index, we evaluated the agreement of experts’ segmentations and illustrated, with CRAR as example, how to assess their performance in absence of a ground truth for comparison. With the help of the developed framework we demonstrated that our method CRAR provides results which are in the range of those of the experts, but with a lower variability. In addition, we confirmed this outcome using a modified state-of-the-art evaluation procedure: based on the results of a paired t-test, we could attest a higher precision of CRAR for detecting minute thickness changes of the choroid.

It can be concluded that CRAR provides a consistent, automated, expert-level performance in recognizing and monitoring subtle choroidal changes before a disease can actually manifest itself. Thus, we want to further develop CRAR so that it can be applied in the prevention and observation of several diseases and their respective treatments. In the ongoing research we plan to add children between 4–6 years to our test group and to verify the algorithm’s performance for a time span of three years, in order to gather more information about the influence of age, height, refractive error and axial length on the thickness of the choroid and to exhaustively discuss the results based on our new framework.

All things considered, the proposed validation framework is suitable for analyzing automatic detection algorithms for choroidal thickness changes, but might also be used for other applications, where a ground truth is not available.

## Supporting information

S1 Appendix Background information about CRAR. (PDF)

S2 Appendix Hardware: Hydra-Spectralis. (PDF)

S1 Table Choroidal thickness changes measurements. (ZIP)
