# Supplementary material for: Statistical framework for validation without ground truth of choroidal thickness changes detection
Source: PLoS One. 2019 Jun 28;14(6):e0218776. doi: 10.1371/journal.pone.0218776 (PMC6599222; doi:10.1371/journal.pone.0218776)
Supplement: S1 Appendix — (PDF) [file pone.0218776.s001.pdf]

S1 Appendix - Background Information about CRAR

In CRAR [1] a piecewise rigid strategy [2] is used in order to elastically deform the soft choroid and preserve the rigid characteristics of the surrounding sclera and retina.

In an initialization step, the slices of the reference and template stack $I_R, I_T : \Omega \rightarrow \mathbb{R}$ images are rigidly registered pairwise at the BM, as shown in Fig. 1. With the help of a graph search-based algorithm [3] the BM is accurately segmented and used as shape-reference for the CSI to initialize CRAR. As explained in [1] this is possible because even in longitudinal studies of progressing diseases the shape of the CSI stays comparable to that of the BM in its small curvature and smoothness. Using CRAR, many problems involving unreliable segmentation can be avoided, because the exact position of the CSI is no longer needed. As a consequence, any other segmentation algorithm applied to any scan within the time sequence could be re-implemented as a starting point for comparisons.

Let $\Omega_j \subset \mathbb{R}^2$ be the jth slice of size $m \times n$ pixels in a volume stack $\Omega = \bigcup_{j=1}^{S} \Omega_j \subset \mathbb{R}^3$ of S slices. Utilizing the BM as a reference surface as aforementioned, the volume surrounding the CSI surface is subdivided into partially overlapping 3D cuboidal blocks $\left\{\mathcal{C}_i^s\right\}$ defined over a regular grid of dimension $\mathcal{N} = N \times \bar{S}$ in the O_{xy} plane, as illustrated in Fig. 2. Using a multiresolution approach, a 3D regularized block-matching registration of the CSI is conducted. As the images are aligned to the rigid BM, the displacement corresponds to shifts of the CSI. Thus, it is possible to determine the displacement field around the CSI and to use its outcome to quantify choroidal growth. Since this study focuses on quantitative choroidal thickness changes in longitudinal studies, only shifts in anterior-posterior/z-direction are considered.

Piecewise rigid registration

In CRAR the registration is conceived as a regularized minimization problem with the aim to find a set $\mathcal{U} = \{u_i^s\}$ of blockwise constant transformations u_i^s such that $I_T(p + u_i^s) \approx I_R(p)$ for all $p \in \mathcal{C}_i^s$.

$$\arg\min_{\mathcal{U}} \mathcal{J}[\mathcal{U}], \quad \mathcal{J}[\mathcal{U}] := \mathcal{D}[I_R, I_T, \mathcal{U}] + \lambda \mathcal{R}[\mathcal{U}]. \tag{1}$$

$\mathcal{D}$ is a distance measure that quantifies the similarity between reference I_R and the transformed template image $I_T(p + u_i^s(p))$. The regularizer $\mathcal{R}$, with its corresponding trade-off parameter $\lambda > 0$, ensures certain properties of the transformation. In comparison to the previous version presented in [1], as of now called CRAR-v1.0, some improvements have been done leading to a better performance in the detection of temporal changes, as depicted in Fig. 4: as we analyze changes in the thickness of the choroid within time intervals of at least three months, the (inverse) normalized cross correlation (see Eq. (2)) is a more useful distance measure than the sum square difference (used in CRAR-v1.0) for such matching problems, where tomograms haven not been acquired at same average signal level. Thus, we define

$$\mathcal{D}[I_R, I_T, \mathcal{U}] := -\sum_{s=1}^{\bar{S}} \sum_{i=1}^{N} \frac{\sum_{p \in \hat{\mathcal{C}}_i^s} \left[I_T(p + u_i^s(p)) - \mu_T\right] \left[I_R(p) - \mu_R\right]}{\sqrt{\sum_{p \in \hat{\mathcal{C}}_i^s} \left[I_T(p + u_i^s(p)) - \mu_T\right]^2} \sqrt{\sum_{p \in \hat{\mathcal{C}}_i^s} \left[I_R(p) - \mu_R\right]^2}}, \tag{2}$$

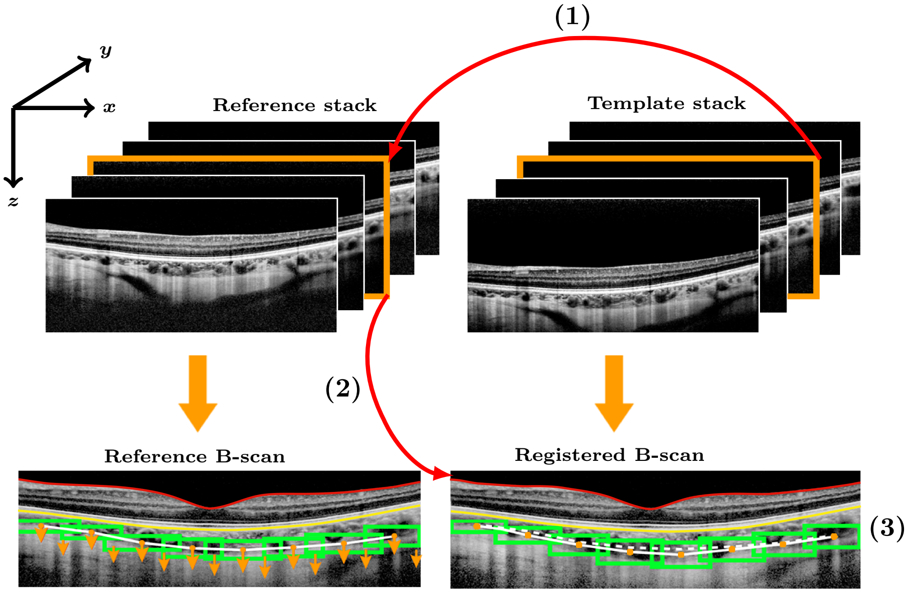

Fig 1. The pairwise inter-stack rigid registration as initialization of CRAR (1), followed by an accurate segmentation of the BM (2). Utilizing the BM as reference, the area surrounding the CSI is subdivided into partially overlapping blocks (3). The displacement is represented by the shifts due to the blockwise transformation of the volume surrounding the lower boundary of the choroid.

where μ_R and μ_T are the average intensities of I_R and I_T respectively, while $\hat{\mathcal{C}}_i^s$ denotes the overlapping volume between reference and transformed template image and p is the point position in block $\hat{\mathcal{C}}_i^s \subset \Omega$.

Radial Differences Regularization

In order to adhere to the eye's natural shape, the regularization enforces the local homogeneity of the transformations in nasal-temporal (x-) and superior-inferior (y-) direction by penalizing their radial differences [4]. The mismatched blocks of the registration process are not individually corrected. Instead, the entire neighborhood is moved until the block configuration with the least bending energy is reached, see Fig. 2. Let $\mathcal{N} = N \times \bar{S}$ be the total number of cuboids, $p_i^s = (x_i^s, y_i^s, z_i^s)$ and $p_j^t = (x_j^t, y_j^t, z_j^t)$ the centers of the blocks $\mathcal{C}_i^s$ and $\mathcal{C}_j^t$, respectively. Then, the regularizer $\mathcal{R}$ is defined as follows:

$$\mathcal{R}[\mathcal{U}] = \frac{1}{\mathcal{N}} \sum_{s,t=1}^{\bar{S}} \sum_{i,j=1}^{N} \left|\left|u_i^s(p_i^s) - u_j^t(p_j^t)\right|\right|^2 \cdot K_b(p_i^s, p_j^t), \tag{3}$$

where $u_i^s(p_i^s)$ and $u_j^t(p_j^t)$ are the corresponding displacement vectors of p_i^s and p_j^t, as obtained from the 3D block-matching. Due to its smoothing properties and compact support, the radial cubic B-spline function $K_b : \Omega \times \Omega \to \mathbb{R}$ has been chosen as kernel. It makes sure that in case of two blocks being wide apart, displacements influence each other much less than if they are within the same $(2\sigma_x \times 2\sigma_y)$-neighborhood. The factor $||u_i^s(p_i^s) - u_j^t(p_j^t)||^2$ of Eq. (3) guarantees local homogeneity of the transformations.

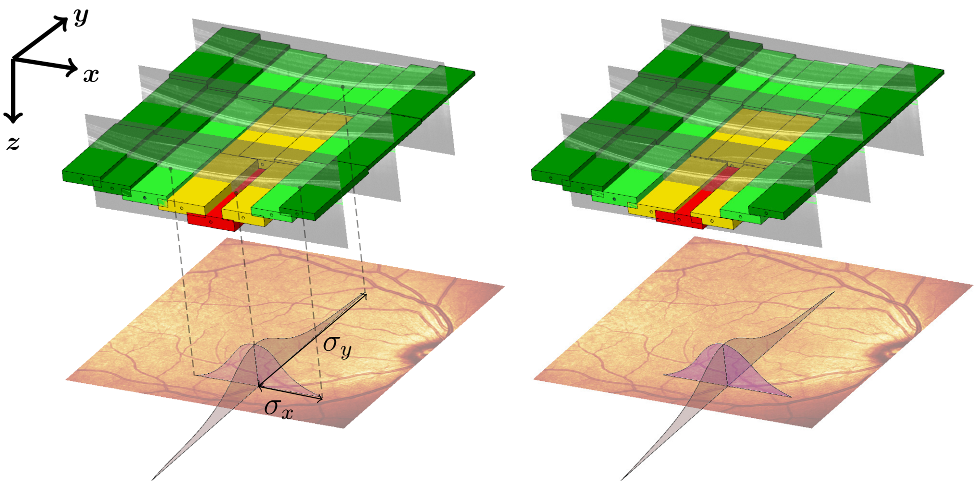

(a) 3D block-matching (b) Additional regularization

Fig 2. (a) Block matching leaves some blocks mismatched (red). Underneath both 1D B-spline kernels with their support $2\sigma_x$ and $2\sigma_y$ are visualized. The regularization corrects the position of the mismatched block in the 3D registration. This movement is counteracted by the directly neighboring blocks (yellow), which has a smoothing effect. Blocks that are further away (light green) do not have such strong influence. (b) The smoothed result after eye shape adherent regularization.

B-spline deformation

Inspired by [5], a synthetically deformed OCT B-scan is created as follows: a target thinning rate α is generated as a random sample from a uniform distribution between $[-25, 25]$ µm, with $\alpha < 0$ denoting a thinning choroid and $\alpha > 0$ corresponding to a thickening one. This range for α is chosen to realistically represent changes that can be observed in the choroid's thickness which are bigger than the daily variations up to 29 µm [6]. A 3D regular B-spline grid with $L_1 \times L_2 \times L_3$ control points is created, see Fig. 3. The region of interest to be manipulated is represented by two rows of grid nodes surrounding the CSI. The B-spline deformation is set to 0 outside this region. In order to attest the superior performance of CRAR in recognizing thickness changes, we apply CRAR on the 90 OCT volume stack pairs after artificially induced deformation [5].

The mean errors in detecting changes by CRAR are compared to those obtained applying the old version of the algorithm, a state-of-the-art segmentation method based

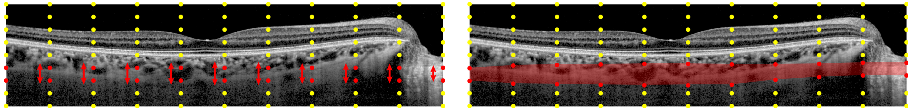

(a) (b)

Fig 3. Artificial deformation of the choroid. (a) A regular $L_1 \times L_2 \times L_3$ grid is created. While the yellow dots remain in place, the red ones surrounding the lower boundary of the choroid are deformed. In thickening simulations (positive factor α), the B-spline grid points in the choroidal region moved away from each other vertically, whereas in thinning simulations (negative α), they moved toward each other. (b) The results after the deformation. The B-spline kernel guarantees local smooth deformations, allowing a realistic thinning/thickening effect.

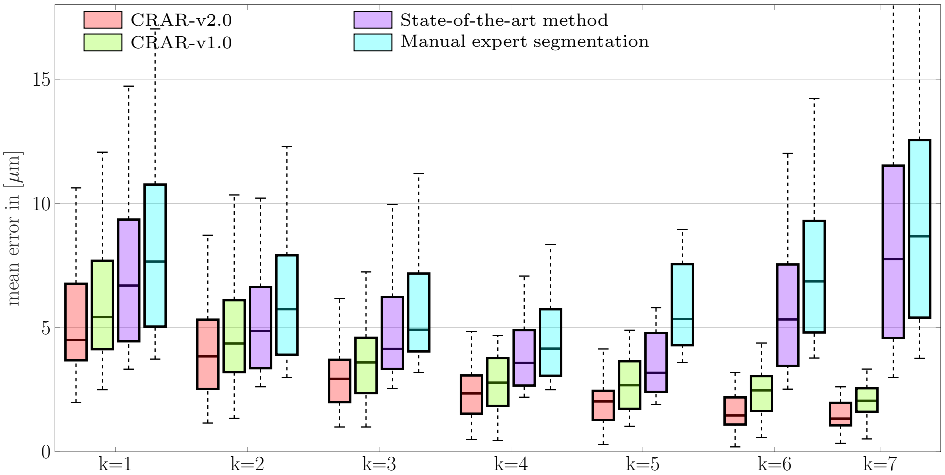

Fig 4. The average differences between the synthetically induced displacements and the measured ones, obtained with CRAR-v2.0 (red), CRAR-v1.0 (green), a state-of-the-art graph search based segmentation method [7] (purple) and manual expert segmentation (cyan), applied on the 90 OCT volume stack pairs after synthetic deformation for different resolution levels k.

on graph search [7] and manual expert segmentation. As illustrated in Fig. 4, we observe that the higher precision of CRAR is remarkable in particular at a higher resolution level. As a reminder, we point out that a multiresolution approach for the registration is used in CRAR (for more detail see [1]). In this context, k denotes the resolution level with the corresponding number of patches in which the volume surrounding the CSI is subdivided in nasal-temporal/x-direction, e.g. $8, 16, \ldots, 512$ for $k = 1, 2, \ldots, 7$. In Fig. 1 the situation for $k = 1$ and 8 blocks is shown.

References

1. Ronchetti T, Maloca P, Jud C, Meier C, Orgül S, Scholl HP, et al. Detecting Early Choroidal Changes Using Piecewise Rigid Image Registration and Eye-Shape Adherent Regularization. In: Fetal, Infant and Ophthalmic Medical Image Analysis. Springer; 2017. p. 92–100.

2. Čech P, Andronache A, Wang L, Székely G, Cattin P. Piecewise rigid multimodal spine registration. Bildverarbeitung für die Medizin 2006. 2006; p. 211–215.

3. Chiu SJ, Li XT, Nicholas P, Toth CA, Izatt JA, Farsiu S. Automatic segmentation of seven retinal layers in SDOCT images congruent with expert manual segmentation. Optics express. 2010;18(18):19413–19428.

4. Jud C, Möri N, Bitterli B, Cattin PC. Bilateral Regularization in Reproducing Kernel Hilbert Spaces for Discontinuity Preserving Image Registration. In: International Workshop on Machine Learning in Medical Imaging. Springer; 2016. p. 10–17.

5. Oguz I, Abramoff MD, Zhang L, Lee K, Zhang EZ, Sonka M. 4D Graph-Based Segmentation for Reproducible and Sensitive Choroid Quantification From Longitudinal OCT Scans4D Choroid Segmentation from Longitudinal OCT Scans. Investigative ophthalmology & visual science. 2016;57(9):OCT621–OCT630.

6. Chakraborty R, Read SA, Collins MJ. Diurnal variations in axial length, choroidal thickness, intraocular pressure, and ocular biometrics. Investigative ophthalmology & visual science. 2011;52(8):5121–5129.

7. Mazzaferri J, Beaton L, Hounye G, Sayah DN, Costantino S. Open-source algorithm for automatic choroid segmentation of OCT volume reconstructions. Scientific Reports. 2017;7:42112.
