# Supplementary material for: Statistical framework for validation without ground truth of choroidal thickness changes detection
Source: PLoS One. 2019 Jun 28;14(6):e0218776. doi: 10.1371/journal.pone.0218776 (PMC6599222; doi:10.1371/journal.pone.0218776)
Supplement: S2 Appendix — (PDF) [file pone.0218776.s002.pdf]

# S2 Appendix - Hardware: Hydra-Spectralis

First of all, we want to emphasize that the OCT-device which we are presenting was used for most of the image acquisition during these studies. However, our algorithm works for any data format, shape, contrast and texture and is therefore not dependent on a particular OCT-device.

The dual-wavelength SD-OCT (Spectral domain/spectrometer-based) system, called Hydra-Spectralis, was developed between 2013 and 2017 at the HuCE-optoLab (optical laboratory at the Institute for Human Centered Engineering) at the Bern University of Applied Sciences (BUAS, Berner Fachhochschule) in Switzerland as result of a cooperation project with involvement of the School of Optometry of the Polytechnic University of Hong Kong, the ARTORG (University of Bern), the Department of Biomedical Engineering (University of Basel) and the Zhongshan Ophthalmic Centre in Guangzhou and was funded by the SNSF (Swiss National Science Fundation) and the NSFC (National Natural Science Foundation of China).

The Spectralis system's base hard-, firm- and software originates from Heidelberg Engineering (Germany) was extended with a second broadband light source that operated at the second ophthalmic NIR window at 1075 nm, a completely reconstructed OCT-light-path and an additional spectrometer, which was developed at the optoLab. Furthermore, the acquisition, tracking and B-Scan registration firm- and software was rewritten to support the dual synchronous channels. The Spectralis is already a full-fledged commercial and clinically approved integrated 870 nm-OCT (Optical Coherence Tomography) device, harbours a coaxial CLSO (Confocal Laser-Scanning Ophthalmoscope) that drives an active, real-time eye-tracker.

The scan-head and its light path with the original wavelength of 870 nm was modified and extended to support the additional wavelength of 1075 nm for simultaneous imaging at both wavelengths. Longer wavelengths are less likely to be scattered by small particles or inhomogeneities as well as changes of refractive index as found in biological tissue. Henceforth, the weaker 1075 nm transparency window of water enables to transfer the radiation deeper into and out of biological tissue without inadvertent scatter from the linear propagation. Therefore, signals returning from deeper layers that are hidden under strong scattering tissue, such as the retinal pigment epithelium and the blood-filled choriocapillaris and choroid, correspond better to the actual biological structures, rather than to shadows or other image artifacts like the 870 nm signals. The choroid can also carry highly scattering melanin, at lower concentration than the RPE. It is more likely that signals from deeper layers are less affected and therefore more clearly represent the true internal structure than at the 870 nm spectrum.

The Hydra-Spectralis system allows to acquire stabilized high-definition cross-sectional images (Brightness-mode or B-scans) of the choroid at both wavelengths. Scattering and absorption reduce the signal with increasing penetration depth. On the other hand the OCT-signal features a positive and negative copy that is symmetric around: the zero-delay, which corresponds to the matched length of the object and reference path. If the reference arm is elongated and the zero-delay is positioned on the side of the weaker signal stemming from deeper regions, the two effects can balance each other. This inversion of the symmetric signal is also referred to as enhanced depth imaging (EDI) and efficiently counteracts the sensitivity fall-off due to scattering with the sensitivity increase close to the zero delay.

It does not distinguish between image artifacts, such as the scattered background seen at shorter wavelengths and real signals, though. Tomograms have been acquired with both channels simultaneously at the same locations in stabilized condition (eye-tracker activated) with multiple (up to 100) scans at the same position. Due to the eye-tracker, even less compliant subjects that interrupt the acquisition can participate, since the system re-tracks the original position and re-builds the complete set of scans at the same lateral locations even in long term follow-up scans. The long sequence acquisition and registered overlapping has multiple implications for the biological information. First, the signal quality is improved due to the $n \times \log(n)$ rule for signal-to-noise improvement for multiple acquisition. Secondly, the changes in imaging conditions slightly alter the speckle structure of the individual frames that originates from local interference of the lights transverse coherence. Averaging of differently speckled frames thus results in a reduction of the speckle in the final image. Thirdly, small scale tissue variations, i.e. those caused by the heart-beat are averaged out and cause a more reliable base line for identifying long term changes of the tissue.
