# Supplementary material for: Statistical framework for validation without ground truth of choroidal thickness changes detection
Source: PLoS One. 2019 Jun 28;14(6):e0218776. doi: 10.1371/journal.pone.0218776 (PMC6599222; doi:10.1371/journal.pone.0218776)
Supplement: S1 Table — (ZIP) [file pone.0218776.s003.zip › S1_Table/Readme.pdf]

S3 Table: Chroroidal Thickness Changes Measurements:

Explanation of the Supporting Information

The names of the B-scans are denoted as in the following example:

21-5a3815281b9cfd1902eec23a2f74b99d-FixA

21 denotes the B-scan position.

5a3815281b9cfd1902eec23a2f74b99d is the anonymization of the patient's name and other relevant data which, otherwise, might infringe on his or her privacy.

FixA: the ending "Fix" refers to the first of the two measurements within a certain interval of time. In case the ending "Reg" is used, this refers to the rigidly registered rescan. In order to determine the intra-rater reliability, each expert segmented an image three times (therefore the suffix "A", "B" or "C").

In the 6 experts' worksheets the fields with "nan" represent the images which were not segmented by the corresponding expert. Since the algorithm directly provides the changes in the choroidal thickness, we inserted them in the "RegA" line and filled the "RegB,C" and "FixA,B,C" lines with "nan" (see worksheet 7 "CRAR").

Corresponding "Fix" and "Reg" values with an absolute difference >150 micrometers were also replaced by "nan".

Each expert's worksheet consists of 4320 lines = 90 (volume stack pairs) x 2 x 3 (segmentations per image) x 8 (scan positions), including 2400 lines with values and 1920 with "nan".

The number of columns is the same for all worksheets: 769 (768 with values in micrometer, corresponding to the number of A-scans and one for the anonymized file's name).
